# SeqTU: A Web Server for Identification of Bacterial Transcription Units

**DOI:** 10.1038/srep43925

**Published:** 2017-03-07

**Authors:** Xin Chen, Wen-Chi Chou, Qin Ma, Ying Xu

**Affiliations:** 1 College of Computer Science and Technology, Jilin University, Changchun, Jilin, China; 2 Computational Systems Biology Lab, Department of Biochemistry and Molecular Biology, and Institute of Bioinformatics, University of Georgia, GA, USA; 3 BioEnergy Science Center, USA; 4 Center for Applied Mathematics, Tianjin University, Tianjin, China; 5 Broad Institute of MIT and Harvard, Cambridge, Massachusetts, USA; 6 Department of Agronomy, Horticulture and Plant Science, South Dakota State University, Brookings, SD, 57007, USA; 7 BioSNTR, Brookings, SD, USA

## Abstract

A transcription unit (TU) consists of K ≥ 1consecutive genes on the same strand of a bacterial genome that are transcribed into a single mRNA molecule under certain conditions. Their identification is an essential step in elucidation of transcriptional regulatory networks. We have recently developed a machine-learning method to accurately identify TUs from RNA-seq data, based on two features of the assembled RNA reads: the continuity and stability of RNA-seq coverage across a genomic region. While good performance was achieved by the method on *Escherichia coli* and *Clostridium thermocellum*, substantial work is needed to make the program generally applicable to all bacteria, knowing that the program requires organism specific information. A web server, named *SeqTU*, was developed to automatically identify TUs with given RNA-seq data of any bacterium using a machine-learning approach. The server consists of a number of utility tools, in addition to TU identification, such as data preparation, data quality check and RNA-read mapping. SeqTU provides a user-friendly interface and automated prediction of TUs from given RNA-seq data. The predicted TUs are displayed intuitively using HTML format along with a graphic visualization of the prediction.

Transcription units (TUs) are basic functional units that each consist of genes consecutively arranged on the same strand of a bacterial genome and transcriptionally co-regulated under specific conditions^1^,^2^. While TUs have the same meaning of the original definition of operons^3^, the definition of an operon has evolved substantially in the past two decades largely due to the widely used operon databases^4^,^5^,^6^, which tend to generally define an operon as a genomic rather than transcriptomic unit and assume that different operons do not overlap. Although operons so defined can be predicted based on genomic sequence data alone^7^, their true utility has been limited, particularly in functional studies of bacterial cells as it has been widely known that genes in the same operons may not necessarily be always co-regulated^8^, i.e., the expressed portion of an operon is dynamically determined by specific conditions. This clearly calls for methods for identification of TUs revealed by transcriptomic data rather than genome sequence data.

Some computational studies have been published for identification of TUs based on Tiling array and RNA-seq data^9^,^10^,^11^, which has offered new information about the complexity of bacterial transcription and regulation. Compared to operons that have numerous public databases and prediction servers, currently there are no prediction services for TU identification. We have recently developed a web server, *SeqTU*, for identifying TUs in bacteria based on an organism’s RNA-seq data, and the pipeline is shown in Fig. 1. A unique feature of the program is that it consists of an automated trainer for training an organism-specific predictor for TUs from the provided RNA-seq data using a machine-learning approach. The underlying algorithm is summarized in the METHODS section and its details can be found in the original algorithm paper^12^. Validation of the program has been carried out on three datasets of *E. coli str. K-12 substr. MG1655* with annotated TUs against predicted ones, and the comparison result suggests that the program is highly reliable.

The predicted TUs are displayed using an intuitive HTML format along with a graphic visualization of the prediction. A link to the HTML page will be sent to the user’s email address once the calculation is done. All users’ computational results will be saved on the server for at least six months. The back-end programs, implemented using PERL and R, along with test examples and documents are freely available online at http://csbl.bmb.uga.edu/SeqTU_dev/index.php.

It is noteworthy that we have previously developed an integrative operon database, DOOR2 (http://csbl.bmb.uga.edu/DOOR/), covering 2,072 bacteria genomes. For each genome, the identified operons and TUs are shown in a genome browser. By integrating the prediction results of *SeqTU* and DOOR2, a user can see the dynamic changes of expressions under different conditions.

## Results

The predicted TUs are summarized in an HTML table, containing the following information: gene name, genomic location, strand orientation, and a read-abundance plot for each nucleotide in a TU. All the results are accessible and more detailed information can be found in the following. The RNA-seq dataset, SRR400619, is used as an example to illustrate how the server can be used to solve a TU identification problem in a bacterial genome.

### Step 1: Data submission

On the homepage of *SeqTU* server (Fig. 2a), a user needs to enter a SRA ID (e.g., the non-strand-specific data SRR400619) to the *search* box; and click on the *next* button below the box. A new page will pop up to collect two pieces of information: (i) the email address for forwarding the prediction results (Fig. 2b), and (ii) the host genome for the given RNA-seq dataset. In this example, only one genome of *E. coli str. K-12 substr. MG1655* is listed in the genome table (Fig. 2c). The user can select this genome, and specify this dataset is not a strain specific RNA-seq data in Fig. 2d. The NC number of the host genome may be required in case the desired host genome is not listed in the genome table (Fig. 2e). Then, the user can click on the *submit* button in Fig. 2e. The server will pop up a page with warning information if an invalid email address is entered. *SeqTU* can automatically download the RNA-seq dataset and the relevant genome sequence and annotation data from the NCBI database based on the submitted information.

To upload a user’s private RNA-seq data from a local machine, one needs to click on the *Submit your own data* button blow the *submit* button (Fig. 2a). The user can upload their RNA-seq data, the host genome along with the anotation files (gff file, ptt file) and the user’s email address in the next page (Fig. 2f).

### Step 2: Job status checking

The user will receive an email immediately after the submission, containing a link to a job-status page. Clicking on this link, the user can find out the job status with the following information: (i) *download progress* of the RNA-seq data download from NCBI; (ii) *quality check* of the RNA-seq data using FastQC. The example SRR400619 has short reads with a length of 36 bps and the sequencing quality is high based on the quality report (Fig. 3a); (iii) *mapping progress* of the RNA reads to the host genome; and (iv) *the final TU prediction* (Fig. 3b). It is noteworthy that if this dataset has been submitted and analyzed previously, this step will be skipped and directly to the next step.

### Step 3: Result page interpretation

When a job is done, the user will be notified by an email with a link to the final TU prediction. In total, 2,329 predicted TUs are listed in the result table of SRR400619. In addition, the start and end positions of each TU on the reference genome, their strand information, gene functional annotations and a download link to read abundance plots are also provided (Fig. 3c). Accuracy scores of each TU prediction are also provided. For example, the TU0005 with a TU prediction accuracy of 0.7813 is composed of *talB* and *mog* genes ranging from 8,238 bp to 9,893 bp on the reverse strand. For the strand-specific example (SRR578142), users can follow the same procedure provided above; and the result table and the TU plot are shown in Fig. 4.

## Discussion

The *SeqTU* server provides a useful and free service for TU prediction from two provided RNA-seq datasets along with the host genomes. A unique characteristic is that the system has a built-in training program that can train an organism-specific classifier for TU prediction. This property makes the prediction highly reliable and desirable. We anticipate that the usefulness of this server will increase over the next few years as more genome-scale transcriptomic data become available and the needs for TU prediction continue to increase.

## Methods

The representation and the logic layers of *SeqTU* are implemented using Web 2.0 technologies (HTML5, CSS3 and Javascript along with jQuery library) and PHP server-side scripting language. It runs on a Red Hat Enterprise Linux 6 box (32 Intel Xeon CPUs with 2.4 GHz and 64GB memory). The input RNA-seq data and the computing results will be stored on a local 10TB hard drive. Considering that a job may take hours up to days to complete, it will not be realistic for a user to sit in front of the computer, waiting for the results to return; hence an email address is collected via the input page, to which the final results will be sent.

**Figure 1:**
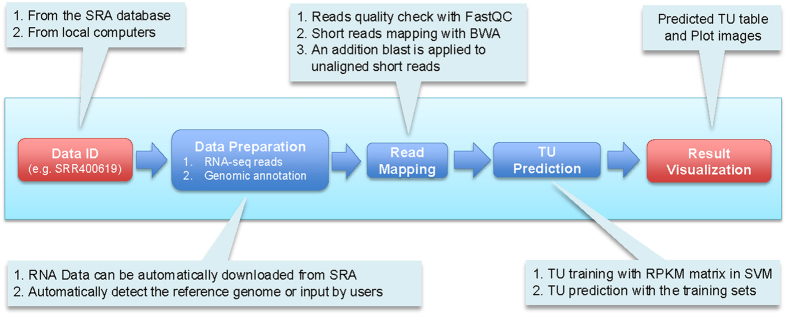
Workflow of *SeqTU server*.

**Figure 2:**
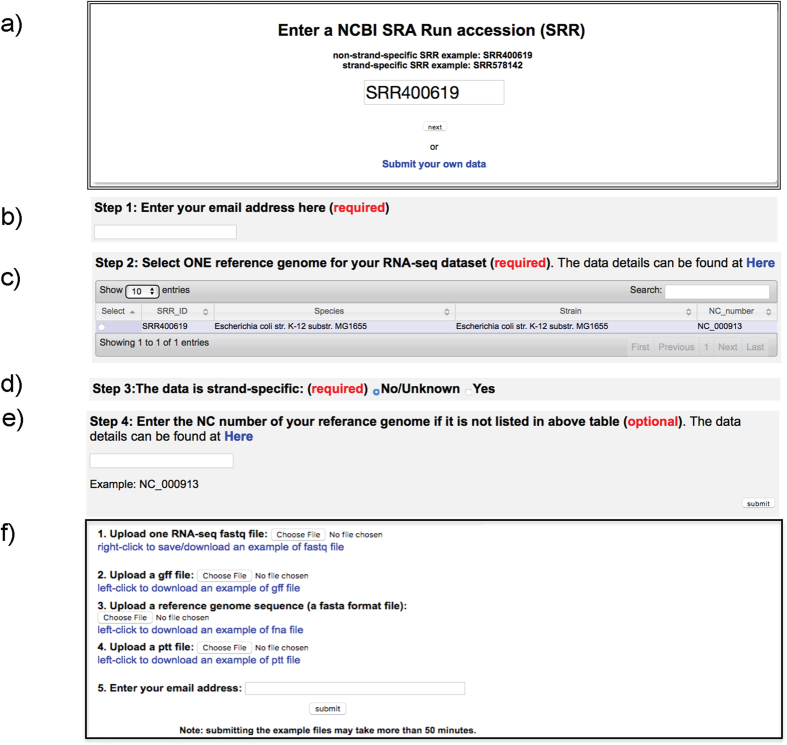
Screenshots of the SeqTU server input pages. (**a**) SeqTU server homepage; (**b**) email input page; (**c**) targeted genome selection page; (**d**) strand specificity option page; (**e**) input NC number of the targeted genome; and (**f**) page for submitting user’s RNA-seq data.

**Figure 3:**
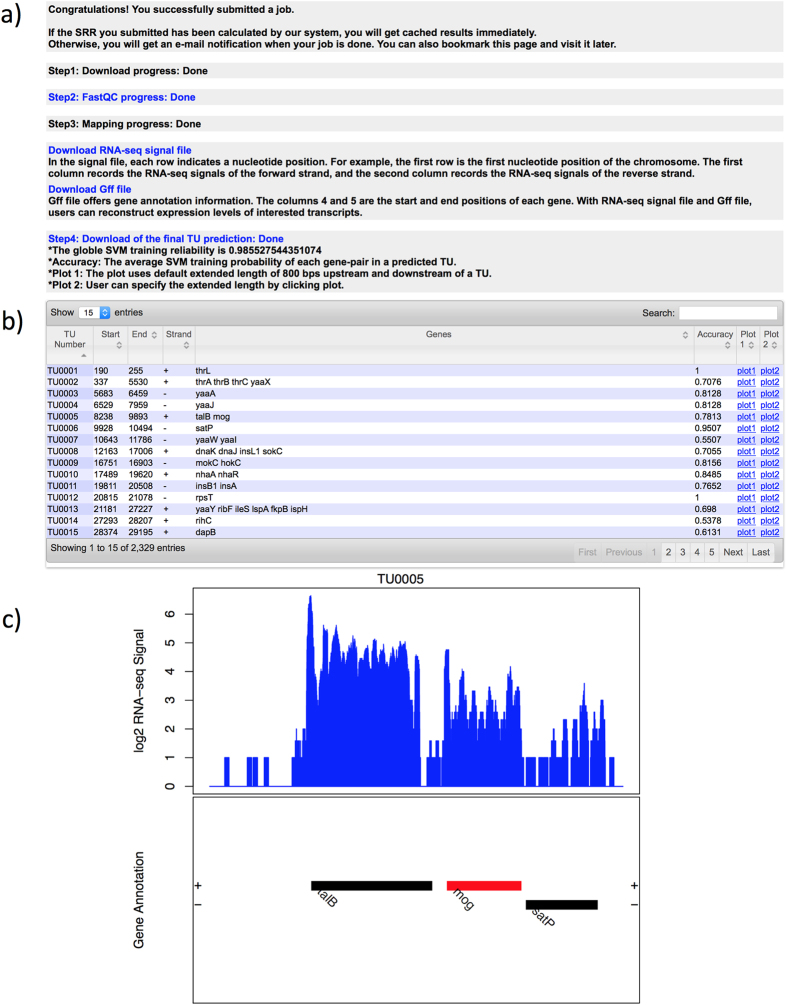
Screenshots of the SeqTU result pages for SRR400619. (**a**) The progress report table; (**b**) The final TU prediction table; and (**c**) An example of computed expression levels over an identified TU in non-strand-specific dataset with default 800 bps upstream and downstream, where the blue histogram represents the read depth over a TU, with the lower part showing the genes in a TU.

**Figure 4:**
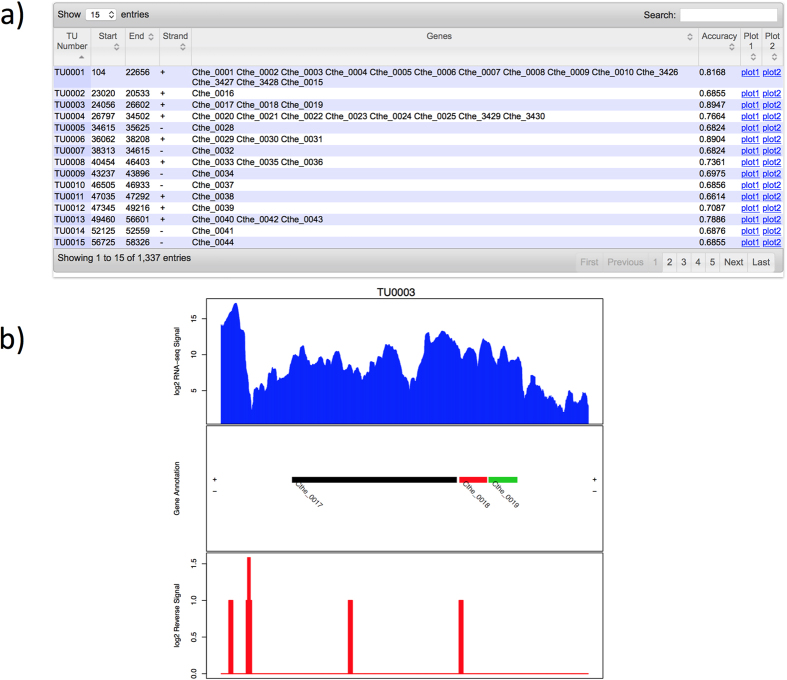
Screenshots of the SeqTU result pages for SRR578142. (**a**) The final TU prediction table; and (**b**) An example of computed expression levels over an identified TU in strand-specific dataset, where the blue histogram represents the read depth over a TU in forward strand, with the middle part showing the genes in a TU, and the red histogram represents the read depth over a TU in reverse strand.

*SeqTU* consists of four main functions: (i) *data preparation*, (ii) *RNA-read mapping*, (iii) *TU prediction,* and (iv) *result visualization* (Fig. 1), as detailed below.

### Data preparation

A user can submit his/her RNA-seq data in one of the two ways: (i) select a dataset from the NCBI SRA database using the ID of the dataset, e.g. SRR400619; or (ii) upload a RNA-seq dataset from the local computer. For now, we restrict the maximum data size for upload to 500MB. In cases where a user’s data size is larger, special arrangements can be made with the developer to allow uploading the dataset in multiple fragments, which will be integrated into one and stored in a temporary location at the server side. For an organism needed for TU prediction, the user can upload the reference genome to the server or select it from the genome list provided by the server.

### Read mapping

Before mapping the RNA reads to the target genome, quality check on the uploaded RNA-seq reads will be performed using FastQC (http://www.bioinformatics.bbsrc.ac.uk/projects/fastqc/), with the check results shown on the result page. The Bowtie2 program^13^ is used to map RNA-seq reads to the relevant genome, and bedtools^14^ will be used to provide the genome coverage information by the uploaded RNA-seq reads, where the genome coverage is normalized by total-sum per million for cross- sample comparisons.

### TU prediction

TU prediction has two steps: (i) training of an organism-specific classifier for TU prediction; and (ii) TU prediction by the trained classifier based on the given RNA-seq dataset. A highly effective method was developed to prepare for positive and negative datasets for training a TU predictor^12^. Considering that experimentally validated TU data are generally lacking, the expressed genes of at least certain lengths and with certain characteristics in their expression pattern are used to mimic two consecutive genes in the same TU and are used as the positive training data. The negative training data consist of consecutive gene pairs deemed to be not in the same TU based on the following criteria: (a) the gap region(s) accounting for more than 50% of the intergenic region between the two genes; and (b) the average expression levels of the two genes are at least 10 fold different, where a gap is defined as a continuous genomic region without any expressions.

A TU classifier is trained based on the given RNA-seq data as follows: an expressed TU should have all its genes expressed at a relatively stable level continuously across the whole TU. The detailed characteristics in stability and continuity of gene-expression patterns of true TUs are learned by the classifier through training on both positive and negative data. A support vector machine-based classifier is trained to distinguish the positive training data from the negative ones. A five-fold cross-validation is used to ensure the classifier is reliably trained. All these are done in an automated manner after the input data file is uploaded. After the training is done, the classifier is applied on the input RNA-seq dataset to generate the final result.

Currently, there are no other programs or web servers can do the same thing as *SeqTU*, so we have evaluated its performance using multiple RNA-seq datasets from different species. The test results of this strategy for calling TUs in *E. coli* and *Clostridium thermocellum* suggest that this strategy works very well^12^. Specifically, on three sets of RNA-seq data^15^ of *E. coli str. K-12 substr. MG1655,* SRX315217, SRX315218, and SRX315219, the trained TU predictors have the prediction accuracy at 0.95, 0.94, and 0.93, respectively. In all the *Clostridium thermocellum* RNA-seq datasets, the trained predictors achieved prediction sensitivities over 0.9. The detailed results can be found in the original algorithm paper^12^.

## Additional Information

**How to cite this article:** Chen, X. *et al*. SeqTU: A Web Server for Identification of Bacterial Transcription Units. *Sci. Rep.* **7**, 43925; doi: 10.1038/srep43925 (2017).

**Publisher's note:** Springer Nature remains neutral with regard to jurisdictional claims in published maps and institutional affiliations.
